# Design of a New Stress Wave-Based Pulse Position Modulation (PPM) Communication System with Piezoceramic Transducers

**DOI:** 10.3390/s19030558

**Published:** 2019-01-29

**Authors:** Aiping Wu, Sihong He, Yali Ren, Ning Wang, Siu Chun Michael Ho, Gangbing Song

**Affiliations:** 1 National Demonstration Center for Experimental Electrical & Electronical Education, Yangtze University, Jingzhou 434023, China; wuaping@yangtzeu.edu.cn; 2 Department of Mechanical Engineering, University of Houston, Houston, TX 77204, USA; she13@uh.edu (S.H.); smho@uh.edu (S.C.M.H.); 3 School of Computer Science, College of Computing, Georgia Institute of Technology, Atlanta, GA 30332, USA; yren78@gatech.edu

**Keywords:** stress wave, stress wave-based communication, piezoceramic transducer, pulse position modulation

## Abstract

Stress wave-based communication has great potential for succeeding in subsea environments where many conventional methods would otherwise face excessive difficulty, and it can benefit logging well by using the drill string as a conduit for stress wave propagation. To achieve stress wave communication, a new stress wave-based pulse position modulation (PPM) communication system is designed and implemented to transmit data through pipeline structures with the help of piezoceramic transducers. This system consists of both hardware and software components. The hardware is composed of a piezoceramic transducer that can generate powerful stress waves travelling along a pipeline, upon touching, and a PPM signal generator that drives the piezoceramic transducer. Once the transducer is in contact with a pipeline surface, the generator integrated with an amplifier is utilized to excite the piezoceramic transducer with a voltage signal that is modulated to encode the information. The resulting vibrations of the transducer generates stress waves that propagate throughout the pipeline. Meanwhile, piezoceramic sensors mounted on the pipeline convert the stress waves to electric signals and the signal can be demodulated. In order to enable the encoding and decoding of information in the stress wave, a PPM-based communication protocol was integrated into the software system. A verification experiment demonstrates the functionality of the developed system for stress wave communication using piezoceramic transducers and the result shows that the data transmission speed of this new communication system can reach 67 bits per second (bps).

## 1. Introduction

Stress wave communication has emerged as a form of wireless communication through solid materials, including metal, rocks, concrete, and among others [1,2,3]. With the increasing need for reliable communication for special applications, such as within concrete structures, well drilling string, metal structures, etc., an increasing number of research works have reported on investigating and applying stress wave communication in many different fields [4,5,6,7]. 

Piezoceramic material, with its unique sensing and actuating capacity [8,9,10], high bandwidth [11,12,13,14], low cost, and wide availability in different shapes and sizes, can be used to build transducers to generate and detect stress waves for a variety of purposes [15,16,17,18], including stress wave-based communication [19] and energy harvesting [20]. Recently, some portable, real-time and wireless systems using piezoelectric transducers have been developed successfully for structural health monitoring (SHM) applications [21,22,23,24,25]. With the piezoceramic transducer as an efficient and economic enabling tool, increasingly, stress wave communications also provide a promising approach to data transmission where the conventional electromagnetic wave-based wireless communication methods are difficult to implement. In the literature, stress wave communication is mainly applied among three types of structures, including concrete structures, metal walls, and pipeline structures.

1. *Concrete structures:* with the development of the embeddable piezoceramic transducers [26,27,28,29,30] researchers have embedded smart aggregate (SA) into concrete structures to build up different communication paradigms mainly using the phase shift keying (PSK) modulation scheme [2,3,6,31].
2. *Metal walls:* through-metal-wall data transmission is crucial for conveying information from within the interior of hermetical containers [32,33,34,35,36]. Stress wave communication is also one of the main techniques being investigated to achieve through-metal-wall data transmission [37,38]. Researchers modulated stress waves with different schemes, such as differential binary phase shift keying (DBPSK) and pulse amplitude modulation (PAM), to achieve through-metal-wall data transmission under different configurations [39,40].
3. *Pipeline structures:* stress wave communication is applied on pipeline structures to achieve data transmission along the pipeline. For example, in a well drilling string, it is critical to obtain the measurements from the bottom of borehole in order to navigate the drilling path. However, as traditional wired communication suffers from practical difficulties such as cable installation and infrastructure adjustment when drilling string sections are added or removed, wireless communication is widely utilized in logging while drilling (LWD) [41]. In the development of stress wave-based LWD wireless communication, the drilling string, which consists of metal pipes and joints, is considered as a conduit for the propagation of information-carrying stress wave [2,42,43,44,45,46]. Compared to the conventional mud pulse telemetry in LWD, stress wave data transmission is less susceptible to the properties of the fluids in the borehole. Meanwhile, stress wave communication is more suitable for underbalanced drilling (UBD) than in mud pulse telemetry or in electromagnetic telemetry [43]. The successful implementation of orthogonal frequency multiplexing (OFDM) based acoustic borehole communication demonstrated the feasibility of stress wave communication through pipeline structures [47,48]. Additionally, other than stress wave-based damage detection and structural health monitoring (SHM) on pipelines [49,50,51], Jin et al. applied stress waves as carriers to transmit information along a steel pipe. Their method was based on time-reversal pulse position modulation (TR-PPM) scheme [52,53]. Furthermore, Trane et al. developed a method for PPM-based communication; however, this method should be applied for communication based on the special multi-wire cables [54,55,56]. Chakraborty et al. proposed an approach to data transmission along a cylindrical pipe [57]. In their work, a simple modulation scheme chirp-on-off keying (Chirp-OOK) is applied, where the bit ‘1’ is represented by a chirp signal with selected frequencies and the bit ‘0’ is represented by a null signal [57,58]. With the implementation of a low-power hardware system, data transmission was successfully achieved. However, due to the effect of channel frequency selectivity [58], the available frequencies for chirp-signals in Chirp-OOK varies with the channels. Additionally, Moll et al. investigated the potential of phase-modulated signals conveying information in a planar waveguide with dispersion compensation based on the guided-wave modes group velocity [59]. A field experiment conducted on a buried water-filled pipe also verified the feasibility of data transmission through an actual pipe [60]. According to the characteristics of a stress wave propagating along a cylindrical pipe, the signal attenuation can undergo steep change with a slight frequency deviation. Thus, PPM is an appropriate modulation scheme for stress wave communication along pipeline structures, since there is no need to consider the available and suitable signal frequency ranges and the waveform refocusing method is verified to be useful to combat the dispersion.

PPM modulation has already been widely applied to the stress wave communication successfully in many different structures, such as pipeline structures, special multi-wire cables, among others. There is still no an easy-to-use system for the PPM modulation stress wave communication, to the authors’ best knowledge. To address this challenge, a new easy-to-use stress wave PPM communication system, including the supporting circuit with a built-in amplifier, a touch type of piezoceramic transducer, and easy-to-use supporting software, is developed in this research in order to achieve data transmission along the pipeline structures. The contributions of our new stress wave PPM communication system are summarized in the following three aspects:

1. To realize stress wave communication, the system integrates PPM modulation circuit and an amplifier to a stress wave generator that can produce high-voltage, modulated, information-containing waveforms.
2. To eliminate the need of an installing device, a new piezoceramic transducer can transmit stress waves through a simple contact with a pipe surface.
3. For the convenience of the end users, an easy-to-use user interface has been designed and implemented. End users can control the data to be transmitted through this easy-to-use interface.

To implement this system, a PPM signal generator, a piezoceramic transducer, and a communication protocol, are designed and implemented. In our design, the piezoceramic generator is utilized to deliver modulated stress wave signals based on the communication protocol. The transducer can be adequately coupled with the transmission medium by a simple contact or touching, thus allowing for flexible usage. The motivation for developing such a piezoceramic transducer is for future robotics applications and the actuator design can be incorporated on a hand of a robot to enable stress wave communication upon “touch.” The rest of this paper is organized as follows. Section 2 illustrates the principle of the stress wave communication system design. Section 3 presents the design process of three key components. Section 4 describes the experiment set up and discusses experimental results. Concluding remarks and future works are summarized in Section 5.

## 2. Principle of Stress Wave-Based Pulse Position Modulation (PPM) Communication

### 2.1. Principle of Stress Wave-Based PPM Communication

A stress wave is the propagation of acoustic waves in solid materials, such as metal, concrete, wood, rock, and among others [61]. Piezoceramic transducers are commonly used to induce the stress wave. Generally, there are three different modes of stress waves: (1) longitudinal waves, also called pressure wave (P-wave); (2) transverse waves, also called shear wave (S-wave); (3) Rayleigh waves, which are a type of surface wave. In Table 1, the velocities of stress wave in different metals are listed [62]. A stress wave traveling in different modes and/or in different metal materials can reach different speeds and distances. Such effects on stress wave propagation will be need to be considered when implementing stress wave communication on metallic pipelines. 

To achieve the data transmission via stress wave through the metal pipeline structures, PPM has been applied in the decoding and encoding process. PPM has been shown to be an effective modulation format for transmitting digital information via acoustic waves because of its suitability for power-efficient channels, comparative simplicity in implementation, and reduced sensitivity to multipath propagation. In PPM, digital information is transmitted by dividing each data frame with duration $\;T_{symbol}$ into M possible data slots with each of duration $\;T_{slot}$, and timing a transmission pulse in one of these time slots. The mathematical definition of a PPM pulse stream is given in [63] as:

(1) $$x\left(t\right)=\;\overset{\infty }{\underset{n\;=\;-\infty }{\sum }}g\left(t-nT_{symbol}-t_{n}\right)$$

where g(t) is the PPM pulse shape and $t_{n}$ is the random data coded into PPM such that:

(2) $$0\;\leq \;t_{n}\;\leq \left(M-1\right)T_{slot}$$

**Figure 1:**
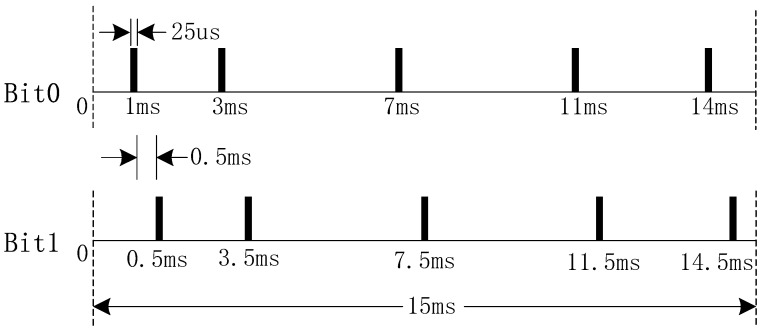
Coding format.

In PPM, data is modulated and transmitted with short pulses all of which have the same width and amplitude. The difference among the pulses is the delay between each pulse. A short duration represents digital 0, and a large duration represents digital 1. In this paper, we applied the following coding format, as shown in Figure 1. The bit ‘0’ is represented by 5 pulses that are set at 1 ms, 3 ms, 7 ms, 11 ms and 14 ms with the frame period of 15 ms and the pulse width of 25  μs. Correspondingly, the pulses standing for bit ‘1’ are set at 1.5 ms, 3.5 ms, 7.5 ms, 11.5 ms, and 14.5ms. After the MCU (microcontroller unit) receives the data from the PC, it will send the data in the format of “55DataAA” to meet the synchronization requirements in demodulation. 

### 2.2. Refocusing Principle

The multi-modal and dispersive characteristics of stress wave propagating along pipes make it difficult to interpret the received waveform correctly. For the purpose of overcoming the ambiguity, correlation analysis is used in an interpreting procedure to refocus the dispersed waveform. If we assume p(t) to be one pulse sequence and h(t) to be the impulse response of channel, then the received signal y(t) can be described as

(3) y(t)=p(t)∗h(t)

where ∗ denotes the convolution operator.

In stress wave based communication, an impulse is first sent to obtain the impulse response before a second signal, which contains the actual data is sent. Therefore: *1*) An initial, single pulse is sent out, and we assume the received waveform as the impulse response h(t) of the channel. *2*) Afterwards, the modulated signal containing the desired information is transmitted and the corresponding received signal is denoted by  y(t). Then, the impulse response h(t) is inverted and convolved with y(t) to refocus the received waveform. The following two equations can represent these two processes:

(4) y(t)∗h(−t)=p(t)∗[h(t)∗h(−t)]

(5) $$G\left(t\right)=h\left(t\right)*h\left(-t\right)=\int_{-\infty }^{\infty }h\left(\tau \right)h\left(\tau +t\right)d\tau$$

In these two processes, the maximum value is reached at  t=0.

## 3. Design of the Stress Wave-Based Communication System

### 3.1. System Architecture Design

**Figure 2:**
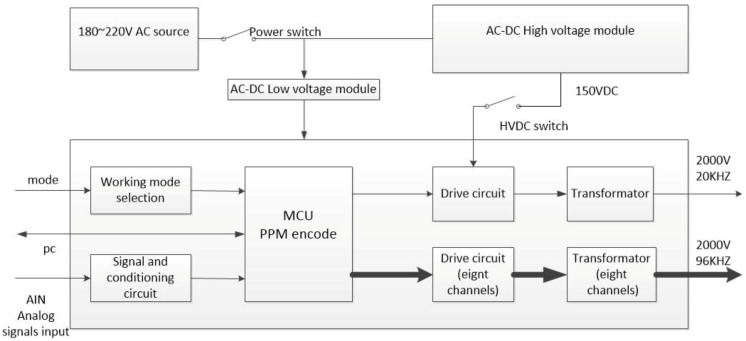
Architecture of the proposed stress wave communication system.

Based on the principles mentioned above, a new stress wave communication system is designed (Figure 2). The proposed system aims to directly generate modulated stress wave carrying information using the PPM. The entire system design process includes two parts, the hardware design and the software design. 

### 3.2. Hardware Design

#### 3.2.1. Piezoceramic Transducer Design

**Figure 3:**
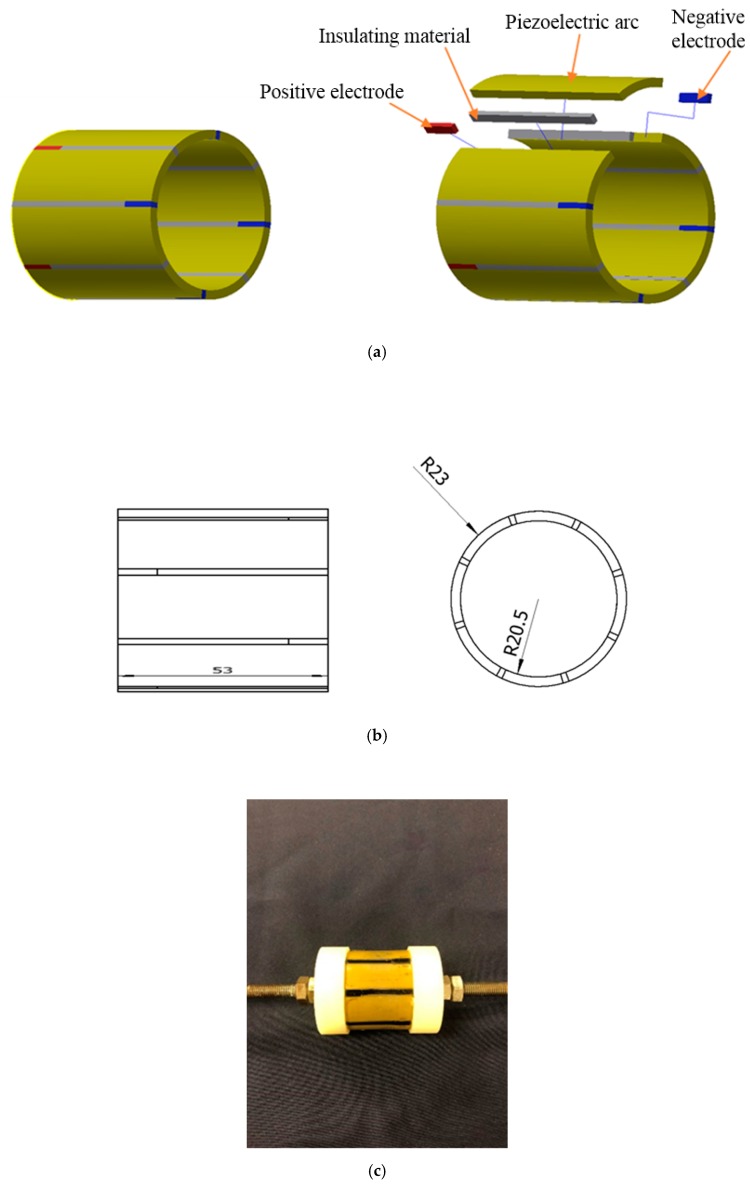
The piezoceramic transducer: (**a**) assembled piezoceramic transducer and components; (**b**) dimensions of the piezoceramic transducer (units in mm); (**c**) photo of the piezoceramic transducer.

The new transducer was designed to generate high-amplitude stress waves upon touching. The motivation for developing such a piezoceramic transducer is for future robotics applications and the actuator design can be incorporated on the hand of a robot to enable stress wave communication upon “touch.” The developed tranducer consists of eight piezoceramic arc segments assembled in parallel to increase vibration amplitude. PZT (lead zirconate titanate), a type of piezoceramic material with strong piezoelectric effect, is used in this research. When excited by an alternating voltage, the eight piezoelectric segments will experience radial deformation. This design of the PZT transducer enables the transmission of the stress wave from the transducer to a pipeline through contact or upon touching. There is no requirement to permanently install the PZT transducer. Table 2 lists the key parameters of the transducer. Figure 3 shows the detailed design of the transducer, which includes the components, dimensions, and a photo. In Figure 3, the red segments represent positive electrodes, and the blue segments represent negative electrodes.

#### 3.2.2. PPM Signal Generator Design

**Figure 4:**
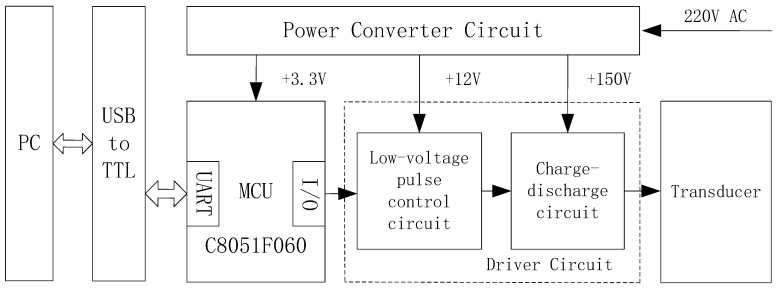
Pulse position modulation (PPM) signal generator design.

As shown in Figure 4, the PPM signal generator is composed of a MCU, a driver circuit, and a power converter circuit. The MCU receives data from the PC, encodes data, and then sends out the encoded data to the driver circuit. The driver circuit combines a low-voltage pulse control circuit with a high-voltage charge-discharge circuit to generate high-voltage pulse signals. The low-voltage circuit controls the high-voltage circuit via an isolation transformer, thereby improving the driving capacity of the metal-oxide-semiconductor field-effect transistor (MOSFET). The high-voltage capacitor and transducer are instantaneously turned on to form up a discharging loop when the MOSFET is turned on, thus actuating the transducer. The power converter circuit converts an input AC voltage into a DC voltage to serve as a low-voltage DC power supply for the MCU control circuit and a high-voltage DC power supply for the driver circuit as well.

##### Microcontroller Unit (MCU) Selection

**Figure 5:**
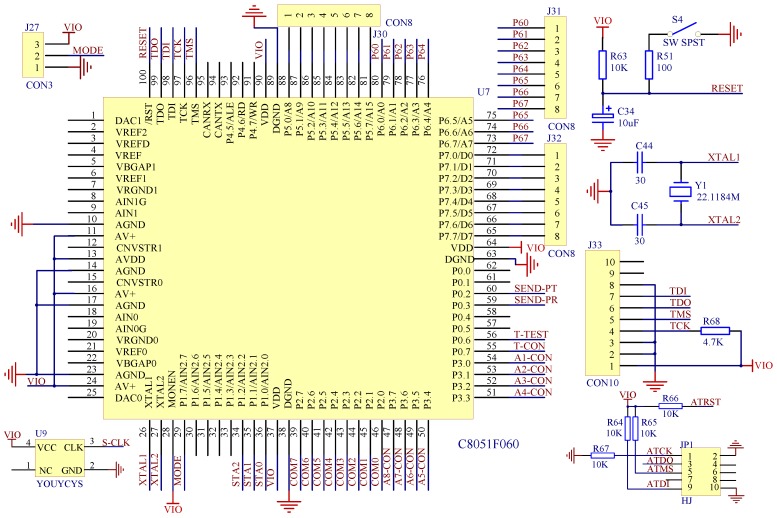
The schematics of microcontroller unit (MCU) control circuit.

The 16-bit single-chip microcomputer C8051F060 is selected for the microcontroller unit, since it features a high-speed CIP-51 core that is compatible with the 8051 microcontroller, an enhanced universal asynchronous receiver-transmitter (UART) serial interface with hardware address recognition for asynchronous serial communication, a general-purpose timer, and a 16-bit analog to digital converter (ADC). As shown in Figure 5, the unit consists of one C8051F060 microcontroller, one clock circuit, one reset circuit and one debug interface. The clock circuit includes C44, C45 and Y1, which uses a 22.1184 MHz crystal oscillator to clock the microcontroller and peripherals. The reset circuit uses a 10 µF capacitor C34 to achieve power-on reset. The port connecting to UART receives data from the PC, the I/O port P0.7 is working as the signal transmission pin to output the encoded data, and the timer T1 is applied while encoding data based on the data transmission protocol.

##### Driver Circuit Design

**Figure 6:**
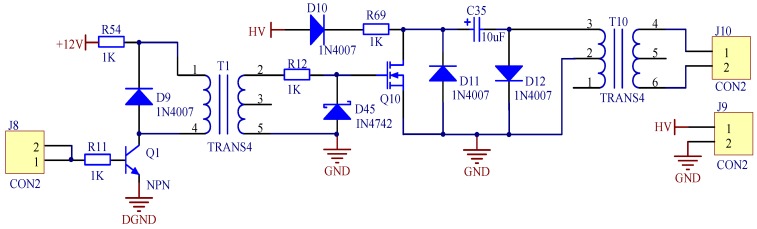
The schematics of the driver circuit.

As shown in Figure 6, the driver circuit consists of a low-voltage pulse control circuit and a charge-discharge circuit. The low-voltage pulse control circuit is composed of an NPN transistor Q2, a transformer T2, and a Zener diode D46. As presented in the left part of Figure 6, if the input pulse voltage at J11 is high, then the transistor Q2 will be turned on, and the current will pass through R55. The current will be generated at the output of T2 due to induction, and then passes through the resistor R13 to drive the MOSFET. The diode D46 acts as a regulator to limit the voltage across R13. On the contrary, if the input pulse voltage at J11 is low, the transistor Q2 is turned off and thereby the driving circuit will not drive the MOSFET.

As shown in the right part of Figure 6, the voltage of DC input at high voltage (HV) is 150 V for the charge-discharge circuit. The output voltage of this circuit needs to be higher than 1000 V to actuate the transducer. Thus, the charge–discharge principle of the capacitor is used to enhance the output voltage. When the MOSFET is grounded, the capacitor C36 will discharge and then a current loop will form up to increase the output voltage of the transformer T1. When the input pulse voltage at J11 is low, neither the MOSFET nor the transformer T1 works. R70 is a power resistor prone to burn out if the power through it is small. The capacitor C36 has a withstand voltage of 600 V.

##### Power Converter Circuit Design

**Figure 7:**
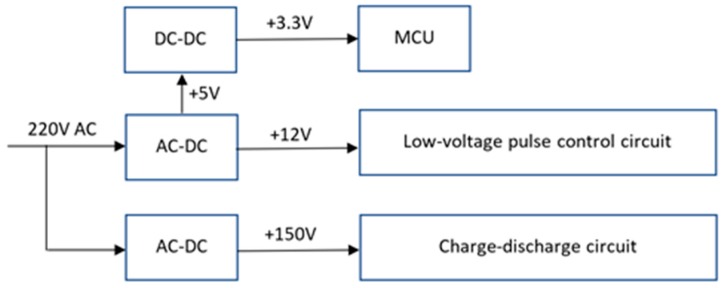
The schematics of power converter circuit.

**Figure 8:**
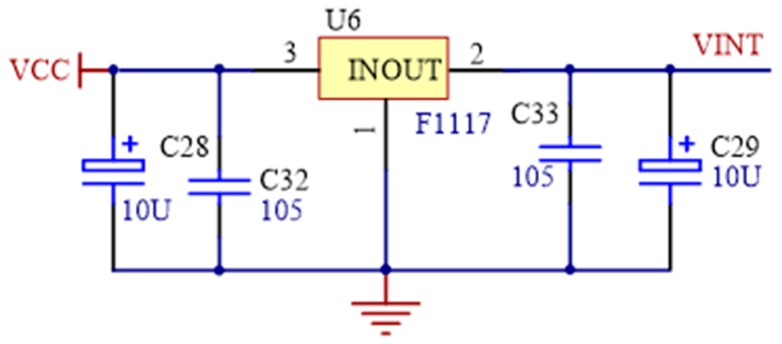
The schematics of regulator.

As shown in Figure 7 and Figure 8, the input AC voltage (220 V) is reduced to ±5 V DC or ±12 V DC by the AC/DC switching power supply module. The +5 V DC is stepped down to +3.3 V via DC-DC to work as the power supply for MCU, and the +12 V DC is used as the power supply for the low-voltage pulse control circuit. On the other hand, another 150 V DC from the AC/DC module serves as the power supply for the charge–discharge circuit.

To satisfy the requirement of power supply for all the components in the device, MEW15-S5D12B was selected for AC-DC switching-mode power supply. It has a broad input voltage range (85~265 VAC, 165~265 VDC), and advantages of low cost, high efficiency, high reliability, and safety. The regulator LM1117 produced by Texas Instruments (TI) was selected for DC-DC buck module to convert +5 V DC to +3.3 V DC. It has current limiting and thermal protection with the input voltage range of 6.2 V~12 V, and the maximum output current of 800 mA.

### 3.3. Software Design

The software system design includes two parts: a main program and a serial interrupt program. The main program is utilized to maintain the sequential execution of each functional module of the system. The serial interrupt program is used to enable the serial port to interrupt after finishing data reception from the PC and sending data back to the PC. To develop the software system, a development tool Keil C51 was used. Keil C51 is a development tool for the 8051 Microcontroller Architecture, and it is designed to solve the complex problems in embedded software system development.

#### 3.3.1. Main Program

**Figure 9:**
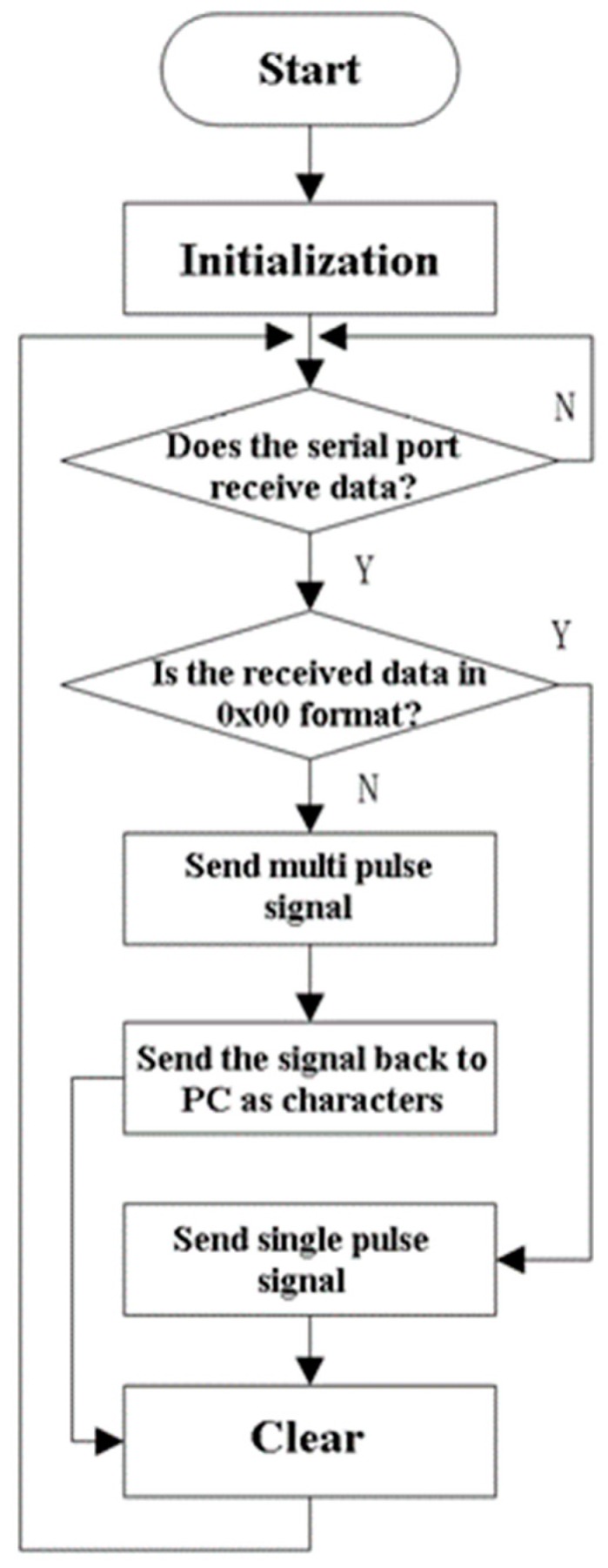
Flow chart of main software program.

The main program handles the following tasks: (1) the initialization of system and peripherals; (2) data reception; (3) stress wave emission; (4) signal return. The flow chart of the main program is shown in Figure 9. 

The initialization is the first step to implementing various functions in the hardware circuit and maintaining the stable operation of the system. After entering the main program, the watchdog is shut down and the system clock is initialized, then the external crystal oscillator is selected as the system clock. As for the initialization of the ports, the I/O port pins are configured according to requirements. Their pins are configured with the I/O mode and assigned to the digital peripherals (serial ports, timers, etc.) by the crossbar configuration register in order of priority. The initialization of the serial ports is mainly about the selection of the working mode, the use of baud rate generator, the setting of baud rate and the interruption itself.

The port pin P0.7 is used to implement the acoustic signal emission in the program. The device emits two types of acoustic signals, single pulse and multi-pulse signals respectively. The single pulse refers to a pulse signal generated by a high instantaneous voltage within 25 µs. The multi-pulse signal refers to a series of pulses that are encoded with the specific coding format, whose shape and amplitude are the same as those of the single pulse. To ensure accuracy, the general-purpose timer, T0 with timing of 25 µs, is selected to cooperate in the generation of pulse signals. The requirements of sending the single pulse, bit ‘0’ and bit ‘1’ are distinguished by the query mode.

#### 3.3.2. Serial Interrupt Program

**Figure 10:**
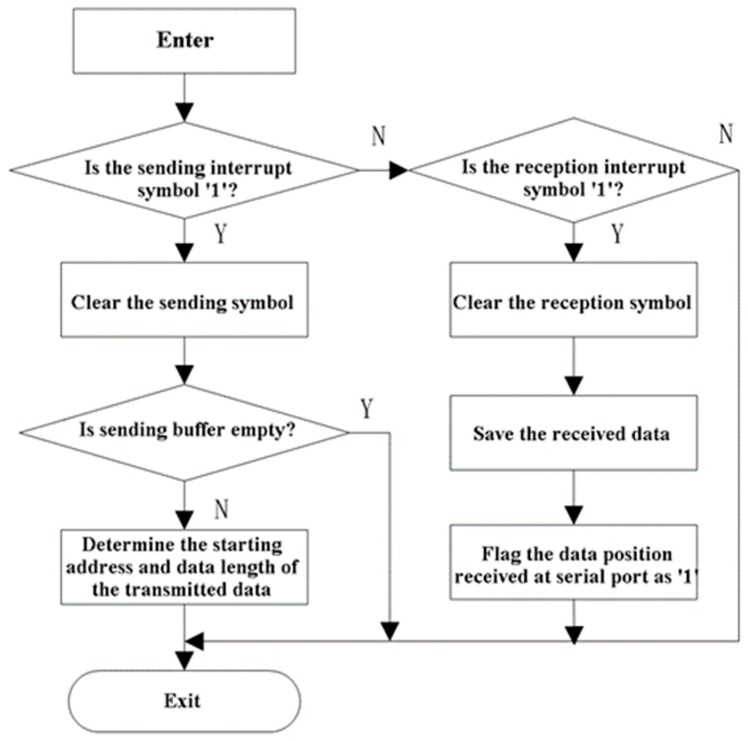
Flow chart of serial interrupt program.

The serial interrupt program handles the data reception and emission. Port 1 is used to complete the communication between the MCU and the PC. Timer 2 works as the baud rate generator of port 1 with the baud rate of 115200 bps. As shown in Figure 10, after entering the serial interrupt program, the first step is to determine the interruption type. If the reception caused the interruption, the program will save the data in a reception buffer and flag the data position received at the serial port as 1. Otherwise, if sending caused the interruption, the program will determine the starting address and byte length of the data, and transmit the data in sequence.

**Figure 11:**
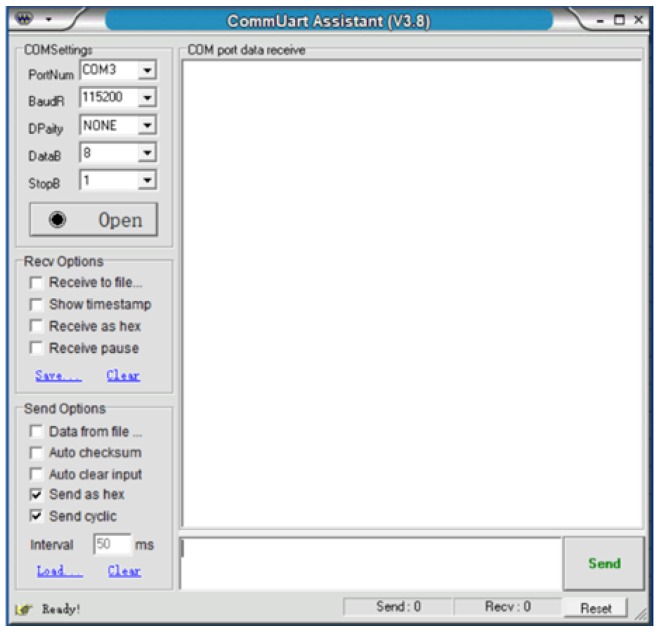
User interface of the stress wave communication system.

Figure 11 shows the software user interface (UI). End users can input the data via the UI, and the input data can be encoded into a hexadecimal sequence and transmitted to the generator.

## 4. Experimental Verification

### 4.1. Experimental Setup

**Figure 12:**
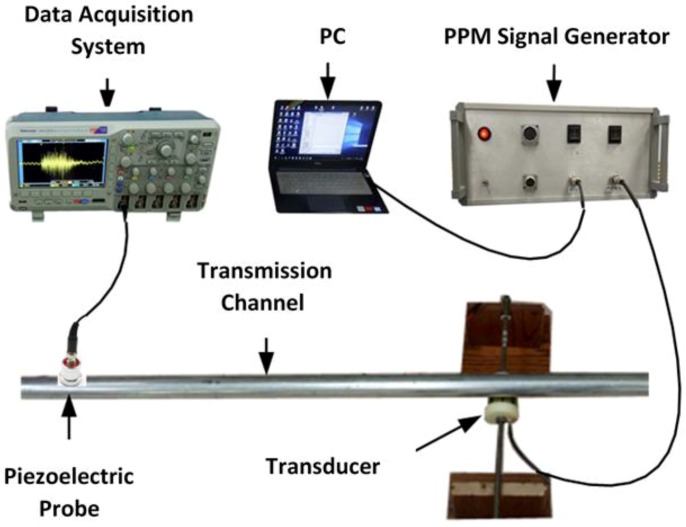
Schematic diagram of the experimental setup.

**Figure 13:**
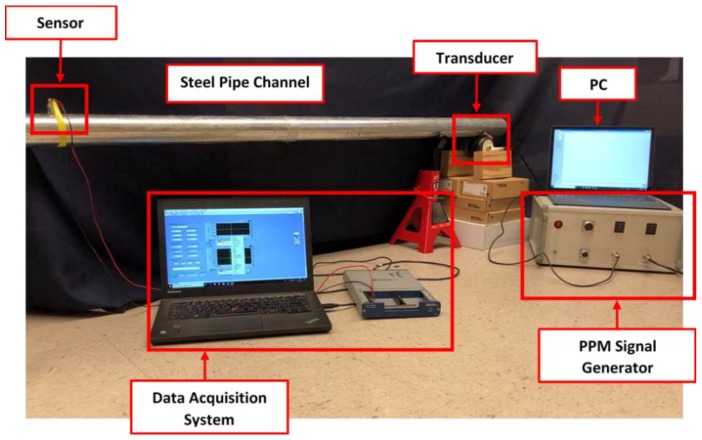
Experimental setup.

As shown in Figure 12, the stress wave communication system consists of a cylindrical piezoceramic transducer, a piezoceramic probe as a sensor, a data-acquisition system, a PC with the communication software, and the PPM signal generator. In the experiment shown in Figure 13, a galvanized steel pipe with an outer diameter of 54 mm and length of 3 m served as the transmission medium. In the setup, the PC controls the transmission of the binary sequence via the USB port, and the generator produces the modulated electrical signals based on the coding format described in Section 4.2. The transducer converts the electrical signals to stress waves that propagate along the pipe, and the piezoelectric probe captures the stress waves. Data from the probe was sampled at 400 kHz. Please note that in the experiment, the developed PZT transducer transmitted the stress wave upon touch since the design of the PZT transducer enables the transmission of the stress wave from the transducer to a pipeline through contact. There is no requirement to permanently install the PZT transducer.

### 4.2. Experimental Results

**Figure 14:**
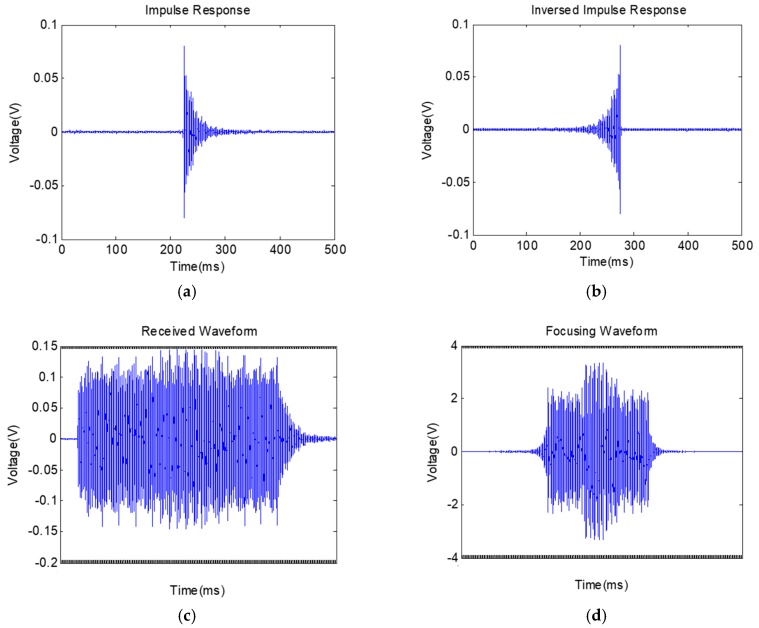

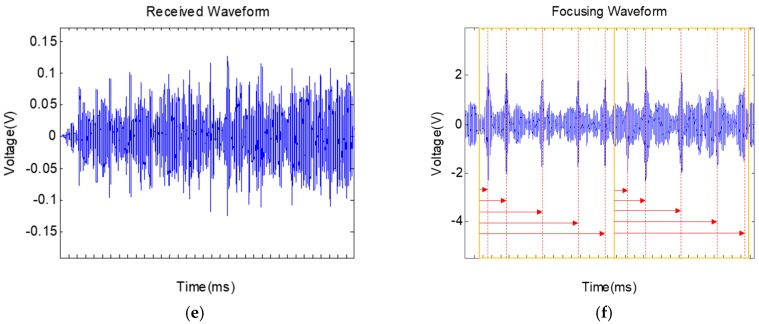
Experimental results: (**a**) impulse response; (**b**) time-inversed impulse response; (**c**) received signal; (**d**) focused waveform; (**e**) zoomed-in figure of received signal; (**f**) zoomed-in figure of the focused waveform.

The impulse response is shown in Figure 14a, and the corresponding reversed impulse response is shown in Figure 14b. One pulse sequence representing [0 1 0 1 0 1 0 1 0 0 0 0 0 0 0 1 1 0 1 0 1 0 1 0] was transmitted through the channel, and Figure 14c shows the received signal. The corresponding focused waveform is shown in Figure 14d. Figure 14e,f show the first 30 ms of Figure 14c,d. From Figure 14f, the received waveform is refocused and the transmitted data can be clearly interpreted. The experimental results verified the effectiveness of this new communication system. Currently, the data transmission rate of the system can reach 67 bits per second (bps) using the stress wave-based transmission.

### 4.3. Discussion

In this section, we verify the feasibility of the stress wave communication system, including both the hardware and the software. In the experiment, we used a galvanized steel pipe as the transmission channel, and the information-containing stress waves were successfully modulated via the software and emitted by the proposed devices. All the received waveforms in the experiments were clearly refocused and interpreted so that we consider the stress wave communication system developed in this paper to have achieved the initial objective. The bit error rate (BER) as a function of the signal-to-noise (SNR) or interference [64,65] requires the investigation of channel characteristics, which will be the focus of our future work on stress wave communication since this paper mainly focuses on the system design. Corrosion, especially that caused by chloride diffusion in subsea evironment, may cause micro damages to the structure and may introduce unwanted disturbance to the communication signals [66,67]. The impact of the structural damages to the stress wave communication signal will be further studied. Since the stress wave can be ued to detect structural damage via the active sensing method [68,69,70,71,72], which is similar to stress wave communication in the sense that a PZT emitter and a PZT sensor are used, we will also explore damage detection by using the stress wave communication signals. 

## 5. Conclusions and Future Work

This paper presented the design of a new stress wave-based PPM communication system using piezoceramic transducers and its verification test. To realize stress wave-based communication, the system integrates a PPM modulation component and an amplifier into a stress wave generator that can produce high-voltage, modulated, information-containing waveforms. To reduce the need for rigid coupling, the new PZT transducer is fabricated into a cylindrical shape that can transmit stress waves through simple contact on a pipe surface. The design of the PZT transducer enables the transmission of the stress wave from the transducer to a pipeline upon touching. Through an easy-to-use user interface, end users can control the data to be transmitted. Finally, the experimental results verified the effectiveness of this new stress wave communication system with the piezoceramic transducer. Currently, the data transmission rate of the prototype system can reach 67 bits per second (bps). In the future, the data transmission rate can be further improved by adjusting the input voltage and output frequency, among other parameters. Furthermore, the proposed system can be easily adapted and applied in future research on stress wave-based PPM communications along pipelines. After fully investigating the channel characteristics, quantitative estimation of the entire system performance will be performed. 

## Figures and Tables

**Table 1:** Velocities of stress wave in different metal materials.

Wave Mode	Wave Velocities (m/s)			
	**Steel**	**Copper**	**Iron**	**Aluminium**
Longitudinal, C	5000	3650	3900	5000
Transversal, $C_{T}$	3200	2250	2450	3050

**Table 2:** Parameters of the piezoceramic transducer.

Name	Parameter
Resonance frequency	17–21 kHz
Static capacitance	1000±20% pf
Electromechanical coupling coefficient	0.52
Operating temperature	−40~200 ℃
Size (outer diameter × inside diameter × height)	53×46×41 mm
